# Shedding of the Salmonid Herpesvirus-3 by Infected Lake Trout (*Salvelinus namaycush*)

**DOI:** 10.3390/v11070580

**Published:** 2019-06-26

**Authors:** Mohamed Faisal, Mochamad Purbayu, Megan A. Shavalier, Terence L. Marsh, Thomas P. Loch

**Affiliations:** 1 Department of Pathobiology and Diagnostic Investigation, College of Veterinary Medicine, Michigan State University, East Lansing, MI 48824, USA; 2 Department of Fisheries and Wildlife, College of Agriculture and Natural Resources, Michigan State University, East Lansing, MI 48824, USA; 3 Comparative Medicine and Integrative Biology, College of Veterinary Medicine, Michigan State University, East Lansing, MI 48824, USA; 4 Department of Microbiology and Molecular Genetics, College of Natural Science, Michigan State University, East Lansing, MI 48824, USA

**Keywords:** Salmonid Herpesvirus-3, Epizootic Epitheliotropic Disease, shedding, lake trout, skin lesions

## Abstract

Salmonid Herpesvirus-3, commonly known as the Epizootic Epitheliotropic Disease virus (EEDV), causes a disease of lake trout (*Salvelinus namaycush*) that has killed millions of fish over the past several decades. Currently, most aspects of EEDV disease ecology remain unknown. In this study, we investigated EEDV shedding in experimentally challenged (intracoelomic injection) lake trout that were individually microchipped. In order to assess viral shedding, each infected fish was placed in individual static, aerated aquaria for a period of 8 h, after which the water was assessed for the presence of EEDV DNA using quantitative PCR. Water sampling was conducted every seven days for 93 days post-infection (pi), followed by additional sampling after one year. Results demonstrated that lake trout began shedding EEDV into the water as early as 9 days pi. Shedding peaked approximately three weeks pi and ceased after nine weeks pi. In contrast, mortalities did not occur until 40 days pi. Although mortality reached 73.9%, surviving fish ceased shedding and continued to grow. However, additional shedding was detected 58 weeks after infection in 66% of surviving fish. Findings of this study demonstrate that EEDV is shed into the water by infected lake trout hosts for extended periods of time, a mechanism that favors virus dissemination.

## 1. Introduction

Herpesviruses are ubiquitous pathogens that infect a wide range of host species extending from mollusks to mammals [1,2,3,4,5,6]. The mechanisms used by herpesviruses to disseminate from one host to another vary considerably. Skin-to-skin contact is essential for the transmission of some herpesviruses, such as Marek’s Disease virus of chickens, which replicates in feather follicles [7], or the Elephant Endotheliotropic Herpesvirus-1 (EEHV-1), which replicates in external trunk cells [8]. Herpesviruses that affect the respiratory system, such as the Equine Herpesvirus-1, are shed from infected bronchi into the air directly [9]. In the case of alloherpesviruses, which infect primarily fish and amphibians, water appears to be the main vehicle for virus transmission [10,11]. Most of our current knowledge on alloherpesvirus transmission stems from two pilot studies. When Yuasa et al. [11] cohabitated common carp (*Cyprinus carpio carpio*) that were previously experimentally infected with Koi Herpesvirus (KHV; Cyprinid Herpesvirus-3) with naïve koi carp (*Cyprinus carpio koi*), the koi carp contracted the virus, suggesting that KHV was transmitted by water. Kancharla and Hanson [10] estimated the duration and level of shedding of the Channel Catfish virus (Ictalurid Herpesvirus-1) in experimentally infected catfish. The authors demonstrated that viral shedding occurred throughout the observation period (12 days) at levels that reached up to 4.5 × 10^7^ copies of viral DNA per fish. 

Among the fish-pathogenic alloherpesviruses, Salmonid Herpesvirus-3, commonly known as Epizootic Epitheliotropic Disease virus (EEDV), causes substantial hatchery losses in its natural host, the lake trout (*Salvelinus namaycush*). Since the late 1980s, this disease has devastated hatchery-reared lake trout populations in the Great Lakes region of North America and caused the demise of millions of fish [4,12,13,14,15]. Despite its high pathogenicity, relatively little is known about EEDV disease ecology in general, and about its transmission in particular. Experimental infection was successful by cohabitation and/or exposure to filtered (450 nm pores) homogenate of infected fish tissues, suggesting water as a likely route of transmission [4,13,16]. Further, using an *in-situ* hybridization assay, [16] it was demonstrated that EEDV targets skin cells more than any of the other external or internal tissues tested. However, the levels of virus shed into the water are currently unknown. Equally unknown is the time frame post-infection (pi) in which infected fish constitute a high risk to other naïve lake trout; i.e., high shedders. To this end, the aim of the present study was to assess the intensity and duration of EEDV shedding in experimentally infected lake trout.

## 2. Materials and Methods

### 2.1. Fish Maintenance

Twenty-nine Lake Superior (LS) strain lake trout (age 25 months post-hatch) were used in this study. The fish were provided by the Marquette State Fish Hatchery (MSFH; Marquette, MI, USA) and maintained at the Michigan State University Research Containment Facility (URCF). Prior to their use in the current study, fish were housed in a 680 L flow-through fiberglass tank supplied with dechlorinated water at 14 °C. The fish were fed AquaMax^®^ Fingerling Starter 300 (Purina^®^, Gray Summit, MO, USA) *ad libitum*, and detritus/uneaten feed was siphoned daily. All fish handling and maintenance was performed in accordance with Michigan State University Institutional Animal Care and Use Committee (IACUC) standards (AUF11/17-197-00, 11/6/2017).

At the start of the study, fish were randomly divided into two groups and placed into separate 42 L cylindrical fiberglass tanks. The negative control group (NC; *n* = 6) received a sham injection, and the experimental group (EEDV group; *n* = 23) was injected with EEDV as described below. The water temperature during this study was set at 10 ± 1 °C in order to closely mimic the water temperature at which natural EEDV outbreaks occur [15]. Fish were allowed to slowly acclimate to the colder water temperatures over a period of 15 days.

### 2.2. Fish Tagging

Fish tagging was performed to allow identification of individual fish throughout the study. A 9 mm- Passive Integrated Transponder (PIT) tag microchip full duplex (FDX; HPT9; Biomark^®^, Boise, ID, USA) was injected into the body cavity of each fish. Before the tagging was conducted, all PIT tag microchips were disinfected by soaking in 70% ethanol for >10 min. Fish were anesthetized with tricaine methanesulfonate (MS-222; Western Chemical, Inc., Ferndale, WA, USA) at a concentration of 0.1 mg/mL water that was buffered with sodium bicarbonate (Church & Dwight Co., Inc., Ewing, NJ, USA) at dose of 0.2 mg/mL. Next, a PIT tag microchip was removed from the 70% ethanol using sterile forceps, rinsed with a sterile phosphate buffered saline solution (PBS; pH 7.5 ± 0.5; Sigma-Aldrich, St. Louis, MO, USA) for 10 s, and placed into the N125 needle (Biomark^®^). Microchip insertion into the body cavity of each fish was performed based on the manufacturer’s instructions for fish >55 mm in length. In order to confirm successful PIT tag implantation, the identification number of each fish was read and recorded using the PIT tag reader (Pocket Reader 098494; Destron-Fearing™, Eagen, MN, USA), after which the fish were transferred back into their respective tanks and observed to ensure proper post-anesthesia recovery. Fish were maintained and observed for 15 days before infection challenges, as described below.

### 2.3. Infection Challenges

A frozen stock of EEDV containing 1 × 10^6^ viral copies/mL was prepared through homogenization of skin tissue collected from naturally infected lake trout as described by Shavalier (2017). For this study, the infectious inoculum was prepared by combining 1.6 mL EEDV stock with 1.4 mL sample diluent (pH 7.525 ± 0.025) containing 458 mL Minimal Essential Medium (MEM; Invitrogen, Thermo Fisher Scientific, Waltham, MA, USA), 7 mL 1 M tris buffer, 1 mL gentamycin sulfate (Sigma-Aldrich), 5 mL penicillin/streptomycin (Invitrogen), and 5 mL Amphotericin B (Thermo Fisher Scientific), resulting in a final working virus concentration of 4.5 × 10^5^ viral copies/mL.

Fish were anesthetized as described above and then injected intracoelomically (IC) with 100 µL/fish of either sample diluent (NC group) or EEDV infectious inoculum (EEDV group). After injection, fish were returned to their respective tanks and monitored daily. Throughout the study, moribund fish were euthanized using an overdose dose of MS-222 (0.25 mg/mL, buffered with sodium bicarbonate at dose of 0.5 mg/mL), after which a gross necropsy was performed. Percent cumulative mortality was calculated by dividing total mortalities through each study period by the starting number of fish in each group (infected and control).

### 2.4. Assessment of Shedding

The twenty-three fish challenged with EEDV were each assigned to one of three sub-groups (1, 2 and 3) for the duration of the study, which consisted of eight, eight, and seven fish, respectively. This division of fish into sampling groups is not expected to have influenced the variables tested in this study. Water sampling was conducted every seven days, on 13 sampling periods, starting at day-7 post-infection (pi) for sub-group 1, day-8 pi for sub-group 2 and day-9 pi for sub-group 3 (Table 1). The NC fish were also assigned to three sub-groups that consisted of two fish each. Fish within a sub-group shared the same water collection day. Each sampling day utilized the fish of one sub-group from the EEDV-challenged fish and the corresponding sub-group of the NC fish. 

On each sampling day, ten 11.4-L glass aquaria (two for the NC group and eight for the EEDV group) were filled with 3.4 L of water (static water system) and placed inside separate 42-L fiberglass cylindrical tanks filled to 18.9 L of chilled water (continuous flow-through system). This arrangement of a static aquarium within a flow-through system ensured a constant sub-ambient (10 ± 1 °C) water temperature in the static system throughout the sampling period. Clean and disinfected air-lines and air-stones were equipped in each glass aquarium to supply appropriate oxygen into the static water system. EEDV-challenged fish and NC fish from a single cohort were selected on each sampling day using a PIT tag reader to identify the PIT tag code and were carefully placed into individual glass aquarium. 

Fish were held in the glass aquarium for a period of eight hours under continuous supervision, after which 40 mL water was collected from the aquarium and stored at −20 °C until DNA extractions could be performed (maximum 2 months after the sample collection date). After the collection, all fish in the glass aquaria were transferred back into their respective tanks (NC tank or EEDV tank). All aquaria, air-lines and air-stones were thoroughly disinfected using 10% bleach (Clorox^®^, Oakland, CA, USA) and/or Nolvasan^®^ (Zoetis Inc., Kalamazoo, MI, USA), prior to their use with the next cohort the following day. It is noteworthy that none of the fish showed signs of discomfort or hypoxia during the period spend in the static aquarium.

### 2.5. DNA Extraction

All DNA extractions were performed following the Alternative PowerSoil Protocol for Low Bacterial Biomass Fluids using the Qiagen DNeasy^®^ PowerLyzer^®^ PowerSoil^®^ Kit (Qiagen, Hilden, Germany) with minor modifications that also included mechanical disruption via bead-beating. Frozen water samples were thawed to room temperature and vortexed briefly. The bead solution (500 µL), phenolchloroform (200 µL; isoamyl alcohol; AMRESCO, Solon, OH, USA), and the C1 solution (60 µL) were added into the supplied bead tubes. 250 µL of the water sample was then added into this mixture, vortexed briefly, and loaded into the bead beater (Mini-Beadbeater-16; Biospec, Inc., Bartlesville, OK, USA) and run on high for 30 s twice with a 20 s rest period between the two bead beating cycles. The mixture was then centrifuged at 10,000× *g* for 1 min at 4 °C. The supernatant was collected and transferred into a new tube provided in the kit and 1 µL of RNase A was added, followed by 100 µL C2 solution and 100 µL C3 solution. Tubes were then vortexed and incubated at 4 °C for 5 min. Samples were centrifuged for 1 min at 10,000× *g* and the supernatant transferred to a new tube. 650 µL C4 solution and 650 µL 100% ethanol were then added to each sample. The remaining steps were followed using the manufacturer’s instructions with the addition of the C6 solution being heated to 60 °C before being used to elute the DNA. Extracted DNA was quantified using a Qubit™ fluorometer (Invitrogen, Eugene, OR, USA), and samples diluted with sterile DNase-free water to a maximum of 12.5 ng/uL qPCR template DNA.

### 2.6. Quantification of EEDV DNA in Water Samples

All qPCR reactions were carried out in a Mastercycler ep realplex^2^ real-time PCR machine (Eppendorf, Hauppauge, NY, USA) and were performed as described by Glenney et al. [17] using the primers 5′-TGG GAG TCC GTC GTC GAA-3′ (SalHV3_23F) and 5′-TCC ACA CAG GAG CTC ACG AA-3′ (SalHV3_23F). Each 20 μL reaction contained 10 μL of SYBR^®^ Select Master Mix, 2 μL of nuclease-free water (Promega, Madison, WI, USA), 1.0 µM of each primer, and template containing 50 nmol total DNA. The qPCR cycling parameters consisted of 50 °C for 2 min; 95 °C for 10 min; and 40 cycles at 95 °C for 15 s, and 60 °C for 60 s. Known EEDV-positive tissue homogenate was used as a positive extraction control (PEC) and sample diluent was used as a negative extraction control (NEC). EEDV-positive purified DNA and nuclease-free water served as the positive reaction control (PRC) and negative reaction control (NRC), respectively. Samples were considered EEDV positive if the fluorescence exceeded 10% of the maximum florescence within 35 amplification cycles as determined with the Mastercycler ep *realplex*^2^ S accompanying software and the manufacturer’s default settings. Positive control standards for quantification were produced using known positive skin samples following the method outlined by Glenney et al. [17]. Shedding rates (viral copies per fish per hour) were calculated using resulting reaction copy number calculated by the Mastercycler ep *realplex*^2^ S accompanying software, sample volume, and sample period length.

## 3. Results

### 3.1. Mortalities and Clinical Signs

**Figure 1:**
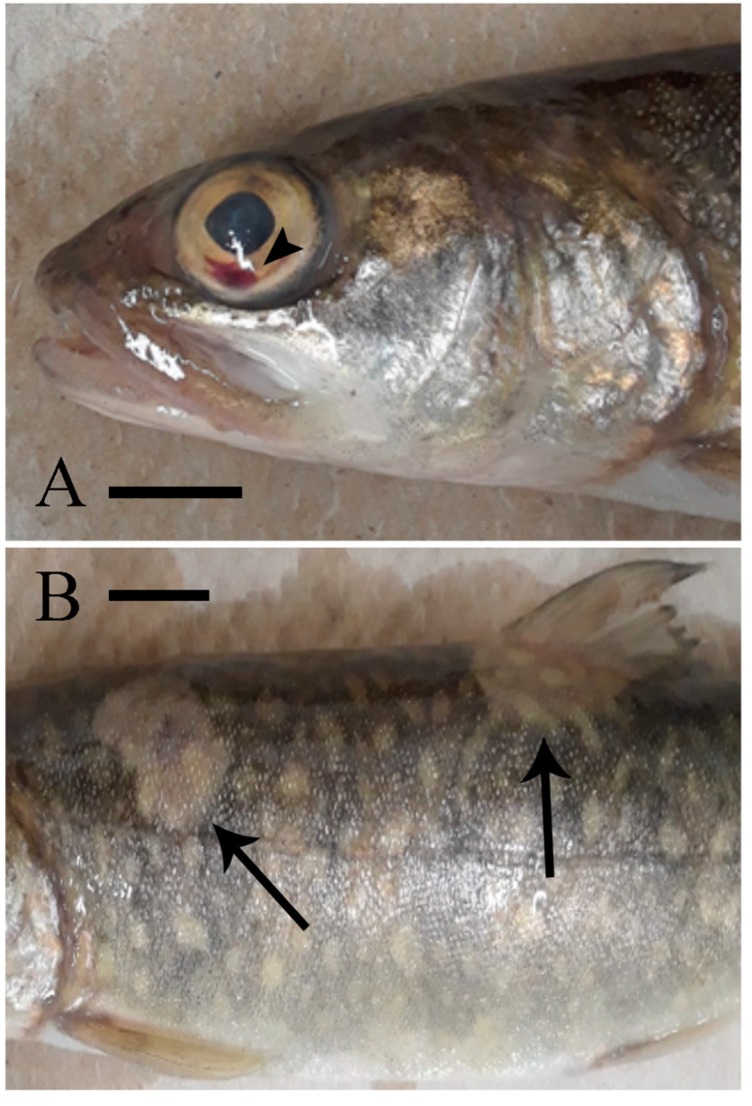
Representative clinical signs of lake trout (*Salvelinus namaycush*) experimentally infected with Epizootic Epitheliotropic Disease virus (EEDV). (**A**) hemorrhage in the lower canthus of the eye (arrowhead) and (**B**) areas of skin pallor and erosion (arrows). Each scale bar represents 1 cm.

**Figure 2:**
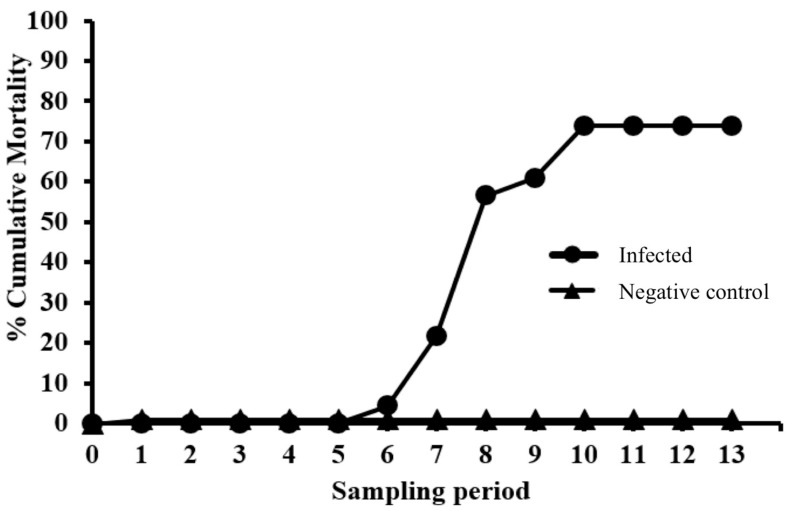
Cumulative mortality (Total mortalities through each sampling period/starting number of fish) of lake trout (*Salvelinus namaycush*) in the Control Group (*n* = 6) and Epizootic Epitheliotropic Disease virus (EEDV) group (*n* = 23) injected with 4.5 × 10^5^ EEDV DNA copies/mL.

Infected fish developed typical signs of EED in the form of hemorrhage in the lower quadrant of the eye (Figure 1A) and focal areas of skin pallor and skin erosions on the trunk (Figure 1B) and around the nares. Mortality in the EEDV-infected group started by day-40 pi and peaked by day-68 pi at 74% (17 of 23 fish) with no additional mortalities through the end of the observation period (day-93; Figure 2, Table 1). External lesions of surviving fish (*n* = 6 fish) healed, and the fish resumed normal behavior and feeding by the end of observation period. No EEDV related clinical signs or mortalities were observed in the negative control fish. 

### 3.2. EEDV Loads in the Water

**Figure 3:**
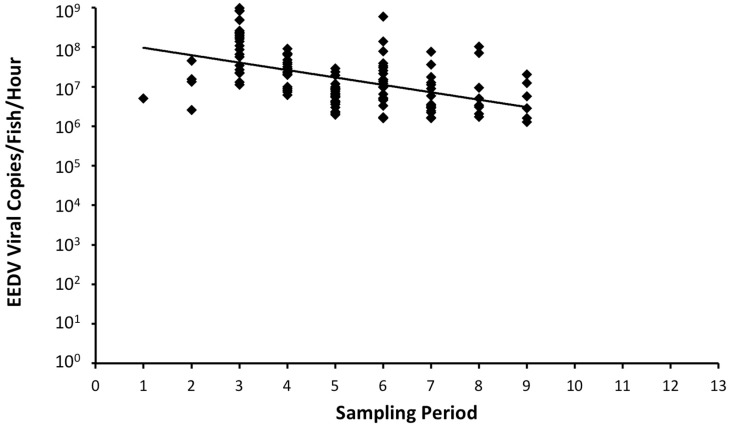
Shedding rate of lake trout (*Salvelinus namaycush*) in the Epizootic Epitheliotropic Disease virus (EEDV) group exposed to EEDV at a dose of 4.5 × 10^5^ viral copies/mL. Each diamond represents the shedding rate of an individual fish. Non-shedding fish are not represented in the graph.

EEDV shedding by infected lake trout, as indicated by the presence of EEDV DNA in the water, started as early as the first sampling period and continued through the 9th sampling period (Table 1, Figure 3). The number of viral shedders increased from 1/23 fish (4.3%) during initial sampling, to 4/23 fish (17.4%) during the second sampling period, to 23/23 fish (100%) during the third sampling period. The percentage of surviving fish that continued to shed the virus remained at or above 80% through sampling period 8, dropped to 66% during sampling period 9 (*n* = 6/9 fish), and dropped to 0% during sampling periods 10–13 (*n* = 0/6 fish).

EEDV shedding rates across all sampling periods ranged from 10^6^ to 10^9^ viral copies per fish per hour, peaking during sampling period 3 (days-21–23) where the average shedding rate was 2.5 × 10^8^ viral copies/fish/hour (*n* = 23/23 fish; Figure 3). Average shedding rates and percentage of fish shedding decreased slightly afterward, and more distinctly beginning with sample period 7 (days-49–51). As surviving fish began to recover from the infection, viral shedding was detected from only 67% of fish at the ninth sampling period (*n* = 6/9; days-63–65), and from no fish at sampling periods 10–13 (*n* = 0/6; days 70–93). 

Shedding was also detected from 4/6 surviving fish 58 weeks post-infection at levels ranging from 2.1 × 10^4^ up to 3.6 × 10^5^. Of the surviving six fish, a subset (*n* = 4) was sampled (fin tissue) and tested negative for EEDV DNA via qPCR. There was no EEDV shedding detected from any control fish throughout the study period.

## 4. Discussion

Findings of this study unravel details on an important aspect of EEDV disease ecology: virus dissemination. Although the virus was injected IC, shedding of EEDV DNA took place in the water and in relatively high titers that far exceeded the initial challenge dose. This implies that initial virus replication took place in the visceral organs, followed by the development of a generalized infection with the virus reaching its target tissue (i.e., skin) as previously reported [15,16]. Shavalier [16] demonstrated the virus’ potential to reach target tissues via the blood stream (viremia), as EEDV was detected in mononuclear cells in the spleen using *in situ* hybridization assay. Closer examination of Figure 3 and Table 1 clearly demonstrates that EEDV needs up to three weeks to reach to the target tissue of the majority of infected fish, at which time high levels of viral replication can lead to skin cell destruction, with sloughed host cells likely facilitating shedding of the virus into the surrounding environment. However, the role of urine, feces, and/or other body fluids in EEDV shedding may also have contributed to the observed shedding loads and warrants further study. 

The detected amounts of EEDV DNA shed per fish per hour were several hundred folds higher than the number of virus copies injected per fish, a matter that likely can overwhelm the immune system of a naïve fish population when cohabitated with a shedding fish. In fact, the hourly virus loads that were shed by individual infected lake trout in this study were substantially higher than the estimated EEDV median lethal dose via immersion previously calculated (i.e., 4.7 × 10^4^ virus copies/mL) [16]. The matter is further complicated by the relatively long time during which high levels of EEDV are shed. Studies done on other fish pathogenic viruses, such as the novirhabdovirus Viral Hemorrhagic Septicemia virus (VHSV), also indicated high levels of shedding that extended up to 15 weeks post exposure [18]. However, the degree of virus amplification by infected fish prior to and while shedding seems to be much higher in the case of EEDV. Whether other Alloherpesviruses have shedding patterns similar to EEDV is currently unknown, since earlier studies were performed using different virus doses, observation periods, endpoints measured, and water temperatures. Of interest, water temperature can have a strong effect on shedding rates of at least one fish-pathogenic herpesvirus, cyprinid herpesvirus-3 (CyHV-3). Yuasa et al. [11] showed that infected fish began shedding CyHV-3 as early as day-7 pi at 16 °C, day-1 pi at 23 °C, and day-3 pi at 28 °C. However, the duration of shedding varied with water temperature, being the longest (34 days pi) at the lowest water temperature (16 °C) and less than half as long at the higher temperatures (e.g., 14 days pi). Water temperature is also important for reactivation of CyHV-3 infections [19] but its effect on EEDV reactivation and/or recrudescence is unknown. In the present study, the water temperature was adjusted to mimic water temperatures measured during natural outbreaks and was constant throughout the observation period.

The sum of our results clearly demonstrated that water is a major vehicle for EEDV shedding; however, how long EEDV can remain infective when kept in water alone (i.e., without fish) remains to be elucidated. In this context, some herpesviruses were found to be relatively stable in aquatic systems. Dayaram et al. [20] showed that equine herpesvirus-1 (EHV-1) maintains infectivity for 14 days in distilled water. Clearly, further studies investigating the length of time EEDV remains infectious in water, both in controlled laboratory environments and under variable field conditions, are needed and will aid future EED prevention and control strategies.

A portion of infected lake trout (~25%) in this study seemed capable of combating EEDV to some degree, whereby they eventually ceased viral shedding and continued to grow. However, fish surviving EEDV infection seem to continue to harbor the virus, since Faisal et al. [15] demonstrated the recrudescence of EEDV in a lake trout population that survived an EEDV outbreak upon exposure to the stress of high rearing density. Similar observations were reported by Eide et al. [21], who failed to detect koi herpesvirus (KHV) in surviving koi fish, yet when these fish were exposed to temperature-induced stress, KHV DNA was detected in gill swabs. Thus, culling of fish surviving an EEDV infection in hatchery populations is likely warranted so as to minimize infection spread.

PIT tagging of EEDV-infected lake trout allowed the identification of individual variations in shedding levels as well as shedding trends. A trend of shedding peaked at ca 3 weeks pi, followed by a decrease in shedding levels that may be due to the demise of most target ectodermal cells and that ultimately ended with host death. Another trend showed that certain fish seemed to better resist and survive the infection despite having high viral loads. The reason for this resistance is currently unknown, primarily because the host immune responses of lake trout to EEDV have not been adequately studied.

In conclusion, findings of this study prove that EEDV is indeed shed from infected lake trout into the water column in high quantities (>10^8^ virus copies/fish/hour) over an extended period of time (≥9 weeks pi). Additionally, individual fish vary in EEDV shedding loads and patterns, whereby some survive initial infection and have the potential to serve as long-term virus reservoirs.

## Figures and Tables

**Table 1:** Epizootic Epitheliotropic Disease virus (EEDV) DNA shedding by experimentally infected lake trout (*Salvelinus namaycush*).

Group	Fish #	Sampling Period												
		1 (Days7, 8, 9)	2 (Days 14, 15, 16)	3 (Days 21, 22, 23)	4 (Days 28, 29, 30)	5 (Days 35, 36, 37)	6 (Days 42, 43, 44)	7 (Days 49, 50, 51)	8 (Days 56, 57, 58)	9 (Days 63, 64, 65)	10 (Days 70, 71, 72)	11 (Days 77, 78, 79)	12 (Days 84, 85, 86)	13 (Days 91, 92, 93)
**Sub-group 1**	1	0	0	2.18 × 10^8^	3.75 × 10^7^	2.90 × 10^7^	*	*	*	*	*	*	*	*
	2	0	0	3.49 × 10^7^	2.36 × 10^7^	9.95 × 10^6^	7.91 × 10^7^	*	*	*	*	*	*	*
	3	0	0	2.37 × 10^8^	2.87 × 10^7^	1.95 × 10^7^	1.51 × 10^7^	0	*	*	*	*	*	*
	4	0	0	8.17 × 10^8^	6.95 × 10^7^	5.87 × 10^6^	3.03 × 10^7^	2.45 × 10^6^	*	*	*	*	*	*
	5	0	0	1.40 × 10^8^	4.82 × 10^7^	7.65 × 10^6^	1.39 × 10^8^	1.76 × 10^7^	1.73 × 10^6^	5.74 × 10^6^	*	*	*	*
	6	0	0	1.13 × 10^7^	6.72 × 10^7^	1.18 × 10^7^	2.57 × 10^7^	7.61 × 10^7^	*	*	*	*	*	*
	7	0	0	2.20 × 10^7^	4.15 × 10^7^	9.08 × 10^6^	2.12 × 10^7^	8.98 × 10^6^	*	*	*	*	*	*
	8	0	4.53 × 10^7^	1.79 × 10^8^	9.09 × 10^7^	3.54 × 10^6^	3.93 × 10^7^	*	*	*	*	*	*	*
**Sub-group 2**	9	0	0	5.64 × 10^7^	3.22 × 10^7^	4.39 × 10^6^	5.90 × 10^8^	3.58 × 10^7^	*	*	*	*	*	*
	10	0	0	1.09 × 10^8^	7.32 × 10^6^	4.21 × 10^6^	3.36 × 10^7^	1.27 × 10^7^	7.03 × 10^7^	1.24 × 10^7^	*	*	*	*
	11	0	0	1.63 × 10^8^	1.97 × 10^7^	8.72 × 10^6^	4.95 × 10^6^	0	1.04 × 10^8^	1.61 × 10^6^	0	0	0	0
	12	0	0	1.98 × 10^8^	6.36 × 10^7^	0	1.62 × 10^6^	1.63 × 10^6^	0	1.29 × 10^6^	0	0	0	0
	13	0	0	4.86 × 10^8^	0	5.41 × 10^6^	5.13 × 10^6^	5.84 × 10^6^	2.04 × 10^6^	2.83 × 10^6^	0	0	0	0
	14	0	0	6.61 × 10^7^	2.09 × 10^7^	2.96 × 10^6^	4.62 × 10^6^	3.09 × 10^6^	0	0	0	0	0	0
	15	0	0	2.70 × 10^7^	0	8.42 × 10^6^	9.74 × 10^6^	*	*	*	*	*	*	*
	16	0	0	2.24 × 10^7^	0	5.61 × 10^6^	1.36 × 10^7^	3.26 × 10^6^	5.02 × 10^6^	2.05 × 10^7^	*	*	*	*
**Sub-group 3**	17	0	0	2.62 × 10^8^	8.03 × 10^6^	3.93 × 10^6^	1.60 × 10^6^	3.60 × 10^6^	*	*	*	*	*	*
	18	0	0	8.66 × 10^7^	9.79 × 10^6^	6.83 × 10^6^	6.53 × 10^6^	1.12 × 10^7^	*	*	*	*	*	*
	19	0	0	1.32 × 10^7^	0	2.18 × 10^6^	3.32 × 10^6^	2.93 × 10^6^	3.09 × 10^6^	0	0	0	0	0
	20	0	1.56 × 10^7^	4.88 × 10^8^	6.20 × 10^6^	1.95 × 10^6^	1.68 × 10^6^	2.22 × 10^6^	9.51 × 10^6^	0	0	0	0	0
	21	0	0	2.36 × 10^8^	2.34 × 10^7^	2.35 × 10^6^	1.53 × 10^7^	1.59 × 10^6^	*	*	*	*	*	*
	22	5.10 × 10^6^	1.34 × 10^7^	9.66 × 10^8^	8.93 × 10^6^	6.45 × 10^6^	1.19 × 10^7^	2.88 × 10^6^	3.42 × 10^6^	*	*	*	*	*
	23	0	2.55 × 10^6^	8.39 × 10^8^	2.85 × 10^7^	2.35 × 10^7^	9.84 × 10^6^	*	*	*	*	*	*	*
**AVERAGE**		5.1 × 10^6^	1.92 × 10^7^	2.47 × 10^8^	3.34 × 10^7^	8.33 × 10^6^	4.83 × 10^7^	1.20 × 10^7^	2.49 × 10^7^	7.38 × 10^6^	0	0	0	0
**STDEV**		0.0 × 10^0^	1.59 × 10^7^	2.76 × 10^8^	2.38 × 10^7^	6.89 × 10^6^	1.22 × 10^8^	1.86 × 10^7^	3.70 × 10^7^	6.95 × 10^6^	0	0	0	0
**# fish died**		0	0	0	0	0	1	4	8	1	3	0	0	0
**# fish shed**		1	4	23	19	22	22	16	8	6	0	0	0	0
**% fish shed**		4.35	17.39	100.00	82.61	95.65	95.65	69.57	34.78	26.09	0	0	0	0

Data are expressed as viral copies/fish/hour. Each sampling period consists of three days and sampling periods are a week apart. “*” indicates fish died. “#” indicates “number”. AVERAGE: the average of fish that were shedding the virus only.
